# Time-resolved trajectory of glucose lowering medications and cardiovascular outcomes in type 2 diabetes: a recurrent neural network analysis

**DOI:** 10.1186/s12933-022-01600-x

**Published:** 2022-08-22

**Authors:** Enrico Longato, Barbara Di Camillo, Giovanni Sparacino, Angelo Avogaro, Gian Paolo Fadini

**Affiliations:** 1 Department of Information Engineering, University of Padova, 35100 Padua, Italy; 2 Department of Comparative Biomedicine and Food Science, University of Padova, 35020 Legnaro, Italy; 3 Department of Medicine DIMED, University of Padova, Via Giustiniani 2, 35100 Padua, Italy

**Keywords:** Artificial intelligence, Prediction, Algorithm, Epidemiology

## Abstract

**Aim:**

Treatment algorithms define lines of glucose lowering medications (GLM) for the management of type 2 diabetes (T2D), but whether therapeutic trajectories are associated with major adverse cardiovascular events (MACE) is unclear. We explored whether the temporal resolution of GLM usage discriminates patients who experienced a 4P-MACE (heart failure, myocardial infarction, stroke, death for all causes).

**Methods:**

We used an administrative database (Veneto region, North-East Italy, 2011–2018) and implemented recurrent neural networks (RNN) with outcome-specific attention maps. The model input included age, sex, diabetes duration, and a matrix of GLM pattern before the 4P-MACE or censoring. Model output was discrimination, reported as area under receiver characteristic curve (AUROC). Attention maps were produced to show medications whose time-resolved trajectories were the most important for discrimination.

**Results:**

The analysis was conducted on 147,135 patients for training and model selection and on 10,000 patients for validation. Collected data spanned a period of ~ 6 years. The RNN model efficiently discriminated temporal patterns of GLM ending in a 4P-MACE vs. those ending in an event-free censoring with an AUROC of 0.911 (95% C.I. 0.904–0.919). This excellent performance was significantly better than that of other models not incorporating time-resolved GLM trajectories: (i) a logistic regression on the bag-of-words encoding all GLM ever taken by the patient (AUROC 0.754; 95% C.I. 0.743–0.765); (ii) a model including the sequence of GLM without temporal relationships (AUROC 0.749; 95% C.I. 0.737–0.761); (iii) a RNN model with the same construction rules but including a time-inverted or randomised order of GLM. Attention maps identified the time-resolved pattern of most common first-line (metformin), second-line (sulphonylureas) GLM, and insulin (glargine) as those determining discrimination capacity.

**Conclusions:**

The time-resolved pattern of GLM use identified patients with subsequent cardiovascular events better than the mere list or sequence of prescribed GLM. Thus, a patient’s therapeutic trajectory could determine disease outcomes.

## Background

Type 2 diabetes (T2D) is a chronic progressive disorder requiring iterated adjustments of pharmacotherapy. The armamentarium for managing T2D has expanded exponentially, and new drugs continue to be released at a fast pace. Furthermore, there is an unprecedented wealth of evidence on safety and efficacy of various glucose lowering medications (GLM) in different populations of patients [1]. With availability of a multitude of treatment combinations, the prevailing concept is that pharmacotherapy of T2D should be tailored to each patient’s characteristics. Scientific societies have issued therapeutic algorithms and guidelines that prioritise certain GLM in specific subgroups of patients while deprioritizing others [2, 3]. This approach necessarily implies a preferred order of GLM and their combinations. For decades, metformin represented the undisputed first-line drug therapy for T2D, but this concept may be changing [3, 4]. Some classes of GLM, such as sulphonylureas, have been repositioned as later options but with considerable heterogeneity among countries and healthcare systems [5]. Initiation of insulin has been moved as a later strategy in most cases [6]. Current algorithms therefore illustrate ideal trajectories that patient should follow according to the available evidence. As evidence and algorithms change over time, many patients with established or long-standing T2D have followed trajectories that would not be appropriate based on today’s knowledge. While therapy can be adjusted to meet a more modern approach, the impact of the prior medication history remains unclear.

SGLT-2 inhibitors and GLP-1 receptor agonists are now considered ideal second-line GLM for most patients with T2D and have strong indications for those with established cardiovascular or renal disease [7–9]. Yet, randomised controlled trials (RCTs) generating such evidence did not test drug positioning along the algorithm as first, second, or more advanced line of therapy. The cardio-renal benefits of these drugs, however, seems to be preserved in patients who were already on sulphonylurea or insulin [8, 9].

Thus, it is uncertain whether the patient’s detailed trajectory in terms of T2D pharmacotherapy can modify disease outcomes. Here, we wished to establish the value of the time-resolved trajectory of GLMs in identifying major adverse cardiovascular events (MACE) among patients with T2D. To do this, we compared the MACE discrimination ability of a deep learning model incorporating a patient’s entire time-resolved pattern of GLM usage versus a model fed by the ordered sequence of GLMs, and another by the list of drugs only. We also challenged the deep learning model with artificial reorderings of the original drug list. Furthermore, we explored the main trends in the relationship between individual GLM patterns and MACE occurrence via the attention mechanism implemented into the deep learning model. We hypothesised that, at means of GLM used in the patient’s history, the ordered, time-resolved patterns of therapy would better discriminate those with incident MACE from those who remained MACE-free relative to what could be achieved by considering either GLM types only or their non-time-characterised sequence.

## Methods

### Data source and study population

The data source used for this study was the administrative claims database of the Veneto region (~ 5 million inhabitants), in Northeast Italy, and, specifically, its prescription medicine and hospital admission repositories with diagnostic discharge codes. Briefly, the Italian healthcare system mandates that all regions collect and share all transactional information on healthcare expenses, including prescription refills and hospitalisations, for reimbursement purposes. As a practical consequence, complete and timestamped information on prescription refills (mapped to ATC codes [10] as per official Ministry tables) and diagnoses at hospital discharge (encoded via ICD-9-CM codes [11]) was available for this study. Additionally, it was also possible to query the regional registry of healthcare beneficiaries [12], to confirm demographics, standing with the regional healthcare system (including month of death), and exemptions from co-payment.

The inclusion criteria for this study were the following: Italian citizenship and residence in the Veneto region; T2D as identified via a validated claims-based algorithm (98% precision, 96% sensitivity) [13]; at least two years of eligibility as per the regional registry of healthcare beneficiaries between 11 January 2011 and 30 September 2018; at least four refilled prescriptions of GLMs (ATC class A10, “drugs used in diabetes”) during the period. Exclusion criteria were: evidence cancer from diagnostic and exemption codes; evidence of prior heart failure, myocardial infarction, or stroke before the start of the observation period.

### Outcome definition and modelling question

The cardiovascular outcome of interest for this study was a version of the 4-point MACE (4P-MACE) composite indicator, defined as the occurrence of at least one between: hospitalisation for heart failure (ICD-9-CM codes starting with 428), myocardial infarction (410–414), or stroke (431–436); or death for any cause.

As previously stated, our objective was to demonstrate whether and to what extent temporal GLM usage patterns, combined with basic information (age, sex, diabetes duration), could identify patients whose trajectories ended on a 4P-MACE. We formalised this task as the following modelling question: “Given the sequence and timing of all GLM prescriptions refilled by a patient (coding resolution: full ATC code; time resolution: trimesters before end-of-observation), and their age, sex, and diabetes duration, does the sequence end on a 4P-MACE, or with the patient’s event-free exit from the database?” Note that this was a classification, rather than temporal prediction, question: in other words, we were only interested in determining where each GLM usage pattern would immediately lead (4P-MACE vs. no event), and not in developing a predicting model to infer something about the future (e.g., survival analysis to determine 4P-MACE probability).

### Data preparation and dataset split

The ground truth for each patient was a set of 5 binary indicators, one for 4P-MACE (primary outcome), and one for each of its components. The 4P-MACE label was equal to 1 if and only if the observed GLM pattern ended immediately before a 4P-MACE, and to 0 in case of event-free exit from the database. Each component label was equal to 1 if and only if the observed 4P-MACE was attributable to that component specifically, and to 0 otherwise (i.e., no 4P-MACE, or 4P-MACE but component not involved); multiple components could be equal to 1 at the same time (e.g., fatal myocardial infarction).

We encoded the pattern of GLMs, plus age, sex, and diabetes duration into a single, 2-dimensional, masked tensor of size 51 features × 25 trimesters. The tensor was also right-aligned, meaning that the j^th^ column (j = 1, …, 25) of the tensor photographed the situation at the (26 – j)^th^ trimester, with the trimester immediately preceding end-of-observation in the last column, and the 25th (6.25 to 6 years before end-observation) in the first column. Observation periods longer than 25 trimesters were cut short by ignoring the oldest data points (26th and earlier trimesters). In case of observation periods shorter than 25 trimesters, the tensor was masked (masking value = –1), i.e., all columns corresponding to unobserved trimesters were uniformly filled with the masking value. Each row of the 2-dimensional tensor encoded age, sex, diabetes duration, or the usage, trimester by trimester, of one of the 48 GLMs available in Veneto at the time of the experiment.

This process resulted in 151,175 2-dimensional tensors, which we split into three subsets: a larger training set for model development comprising the data of 131,175 patients, a validation set of 10,000 patients for hyperparameter tuning (if needed), and a test set of 10,000 patients for final performance evaluation.

### Model architecture and output

Our model is based on the deep recurrent neural network (RNN) architecture proposed in [14], and adapted from the context of clinical event prediction to GLM usage pattern classification. The main feature of both the original and our version of the architecture is its input-level attention mechanism, i.e., the presence of a specific layer that established a relative importance weighting between ATC classes at each time point [15].

**Fig. 1:**
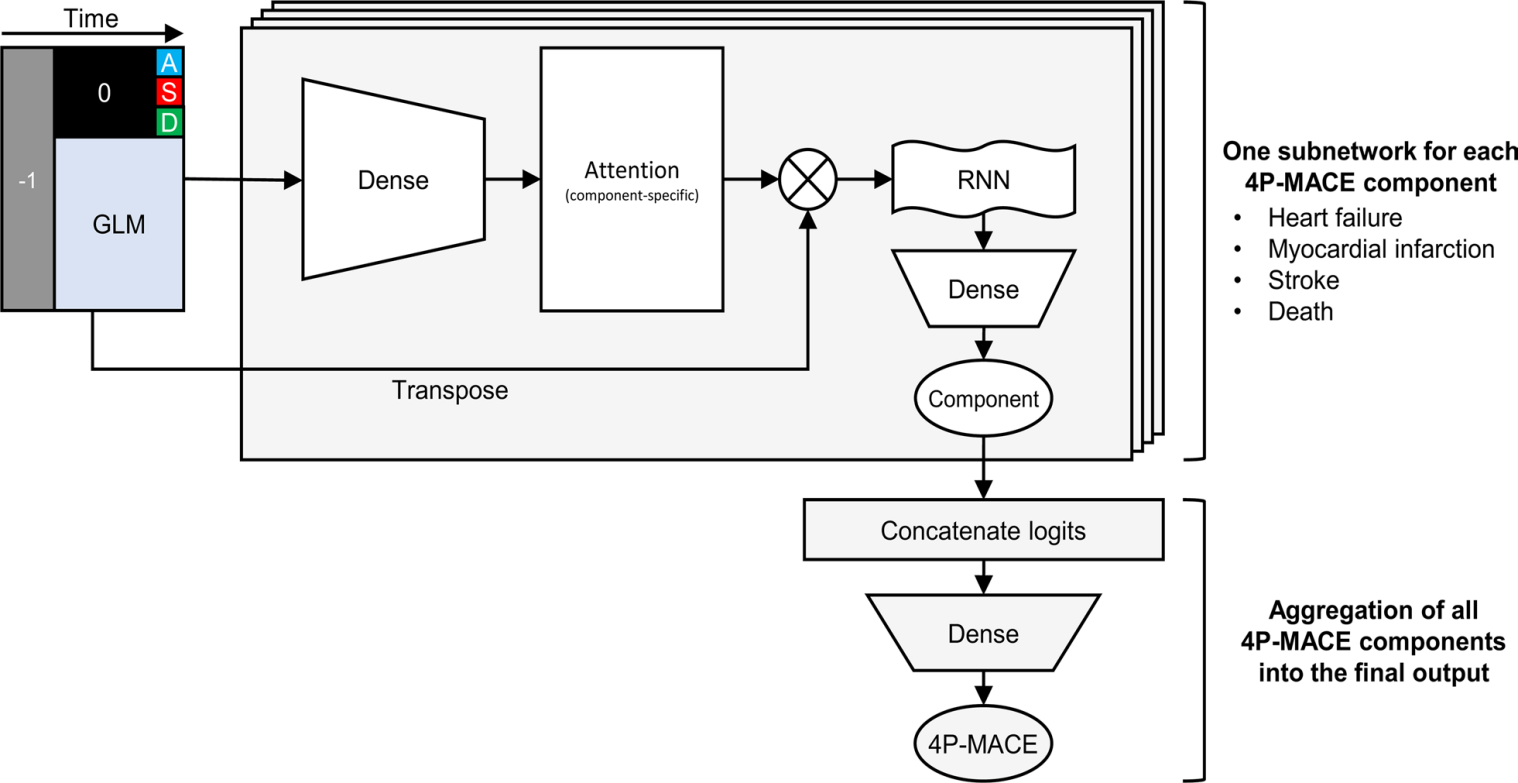
Architecture of the model. GLM, glucose lowering medications. RNN, recursive neural network. *4P-MACE* 4 components of the major adverse cardiovascular event composite outcome

Our model architecture conceptually implements a cascade of four logical steps, namely: tensor ingestion, the attention mechanism, a recurrent layer, final prediction via fully connected layers (Fig. 1). First, the 2-dimensional input tensor is duplicated: one of the copies is passed to the attention mechanism, the other is transposed and ready to be multiplied by an attention matrix. At this point, the network splits into four parallel, identically structured subnetworks, one for each 4P-MACE component. Within each subnetwork, to implement the attention definition used in [14] (plus a bias term) using the computationally efficient tools available within the main deep learning libraries, the first copy of the tensor enters a dense layer of 25 (number of trimesters) neurons equipped with a softmax activation function. This process results in an attention matrix that assigns a weight to each GLM used in each trimester such that the sum over time of the weights is equal to 1, while the sum over all features of the weights attributed to a trimester is unbounded. In other words, for each subject, the network tries to establish the relative importance of each GLM within each trimester and the overall importance of the trimester. After computation, the attention matrix is transposed and multiplied elementwise by the transposed copy of the input two-dimensional tensor, thus implementing the input-level attention mechanism. As there are four subnetworks, we also obtain four (different) attention matrices and four attention-weighted tensors. Each weighted tensor, then, passes through a recurrent layer (a LSTM [16] or GRU [17], possibly with dropout) that squeezes the dynamic, variable-length information carried by the tensor into a single, fixed-length vector. A dense layer with a single neuron and sigmoid activation yields each subnetwork’s output, to be compared to the ground truth of the corresponding 4P-MACE component. Finally, the four subnetworks are brought together via concatenation of the four terminal pre-activation logits, and the resulting 4-element vector is passed to a dense layer with a single neuron, which outputs the final 4P-MACE prediction.

In summary, the model has one primary output, i.e., the score (or probability) associated with the likelihood of an observation window ending on a 4P-MACE vs. on an event-free exit from the database, and four component-specific secondary outputs.

As retrieval of outcome-specific attention maps was possible for all subjects, we produced four average attention matrices, one for each 4P-MACE component. We turned each map into an attention landscape, re-normalised, for legibility, within each trimester, to show the time-resolved patterns of GLMs that most contributed to classification.

### Model selection and primary performance evaluation

Given the architecture described above, we selected the final model via hyperparameter tuning based on an exhaustive grid search and early stopping. We tested 216 hyperparameter combinations: presence or absence of a bias term in the attention mechanism, LSTM or GRU as the type of recurrent layer, 64, 128, 256 as the number of recurrent units, rectified linear unit (ReLu) or hyperbolic tangent as the recurrent layer’s activation function, 0%, 10%, or 25% as recurrent layer’s dropout and recurrent dropout (independently). For each combination, we optimised all model parameters using the average binary crossentropy of 4P-MACE and its components as a cost function (ADAM algorithm, learning rate = 0.001); then, we evaluated the area under the receiver-operating characteristic curve (AUROC) for 4P-MACE on the validation set (10,000 patients not used for parameter estimation), stopping the training process after 10 epochs of no improvement, and retaining the best epoch’s parameters. We selected the best model among the 216 candidates as the one maximising the 4P-MACE AUROC on the validation set.

We evaluated the final model’s performance in terms of the AUROCs associated with 4P-MACE and each of its components on the test set (untouched until this point), including 95% confidence intervals calculated via the DeLong method [18].

### Secondary benchmarking analyses

The proposed model can leverage on the three fundamental aspects of GLM usage (namely, timing, sequence, and type of medication). However, this comes at the cost of having to handle relatively large (51 × 25) input tensors. Hence, to quantify the possible impact of input type and dimensionality on classification performance, we set up one primary and three secondary analyses following the same experimental protocol and data splits used in the primary analysis. The outputs of each analysis were the classification AUROC on the test set, including 95% confidence interval, and the identification of statistically significant difference in performance vs. the proposed model.

First, to understand the impact and efficiency of sequence-based learning with respect to classification performance, we reran the performance evaluation phase on two artificially modified variations of the test set. Namely, we considered a variation where the unmasked portion of the tensor was randomly shuffled through time, and one where the order of refilled prescriptions was completely inverted (we pretended that the first GLM was prescribed at the date of the last GLM, the second of the second-to-last, etc.).

Second, we implemented a strategy adapted from [19], which requires medication data in the form of variable length (hence masked and zero padded to 150 refills) sequences. On the one hand, the switch from tensor to sequences collapsed the “timing” dimension and reduced dimensionality; on the other, it removed the model’s ability to account for simultaneous therapies. In practice, we transformed each tensor into a zero padded (and masked with masking value = 0) sequence of integers (from 1 to 48, each corresponding to a GLM ATC class) sorted from oldest to newest according to their prescription date; and treated age, sex, and diabetes duration as a separate input. We substituted the initial part of the proposed architecture (tensor ingestion and attention mechanism) with the corresponding solution taken from [9], i.e., sequence ingestion, embedding, and concatenation of patient information with the output of the recurrent layer, using the following hyperparameters (216 combinations): learning rate = 0.001, embedding size (64 or 128); recurrent layer type (LSTM or GRU) and number of units (64, 128, or 256), activation function (ReLu or hyperbolic tangent), dropout and recurrent dropout (for both, independently: no dropout, 10%, or 25%). The rest of the pipeline remained unaltered. Note that, at strong variance with the model in [9], here, we tackled a classification (vs. prediction) task, resulting in a much wider observation window of 6.25 years (vs. 1 year), and static (vs. dynamic, 1 to 5 years in the future) ground truth labels.

Third, we developed the simplest possible model, i.e., a logistic regression on the concatenation of age, sex, diabetes duration, and the bag-of-words vector of prescribed GLMs throughout the entire observation period. This analysis further collapsed all information carried by the “sequence” dimension into a static vector of 51 elements.

## Results

### Patient characteristics

The training, validation, and test sets were homogeneous in terms of both baseline characteristics and outcome incidence (Table 1). Patients were on average 45% female, 71 years old, had had diabetes for approximately 11 years, and had 6.4 years of available baseline data. Overall, 21% of GLM usage patterns ended in a 4P-MACE, and, specifically, the cumulative incidence of the non-mutually-exclusive components was 5.4% for heart failure, 6.4% for myocardial infarction, 4% for stroke, and 6.7% for all-cause death.

**Table 1:** Characteristics of the study population

	Training	Validation	Test
N. subjects	137,175 (87.3%)	10,000 (6.4%)	10,000 (6.4%)
Female sex	62,103 (45.3%)	4561 (45.6%)	4484 (44.8%)
Age (years)	71.2 ± 13.5	71.2 ± 13.5	71.0 ± 13.8
Diabetes duration according to claims (months)	131.9 ± 71.9	131.7 ± 72.1	131.2 ± 72.2
N. hospitalised at baseline	55,762 (40.7%)	4052 (40.5%)	4056 (40.6%)
Baseline length (days)	2338.5 ± 86.0	2338.3 ± 87.4	2337.2 ± 86.8
Long-acting insulin	39,566 (28.8%)	2877 (28.8%)	2983 (29.8%)
Fast-acting insulin	29,926 (21.8%)	2195 (21.9%)	2241 (22.4%)
DPP4i	24,656 (18.0%)	1748 (17.5%)	1793 (17.9%)
GLP-1RA	7372 (5.4%)	512 (5.1%)	513 (5.1%)
SGLT2i	6053 (4.4%)	456 (4.6%)	487 (4.9%)
Sulfonylureas	66,412 (48.4%)	4843 (48.4%)	4822 (48.2%)
Ischemic heart disease	9,672 (7.1%)	734 (7.3%)	694 (6.9%)
Pioglitazone	12,379 (9.0%)	879 (8.8%)	893 (8.9%)
Cardiovascular disease	12,108 (8.8%)	915 (9.2%)	876 (8.8%)
Platelet aggregation inhibitors	67,386 (49.1%)	4927 (49.3%)	4853 (48.5%)
Chronic kidney disease	4866 (3.5%)	354 (3.5%)	340 (3.4%)
Statins	82,802 (60.4%)	5996 (60.0%)	5926 (59.3%)
Dyslipidaemia	87,415 (63.7%)	6343 (63.4%)	6271 (62.7%)
Metformin	111,113 (81.0%)	8141 (81.4%)	8049 (80.5%)
Beta blockers	50,873 (37.1%)	3750 (37.5%)	3643 (36.4%)
Other antihypertensives	16,030 (11.7%)	1202 (12.0%)	1176 (11.8%)
Charlson comorbidity index	0.3 ± 1.0	0.4 ± 1.1	0.4 ± 1.0
Ocular complications	611 (0.4%)	52 (0.5%)	41 (0.4%)
ACE inhibitors	98,958 (72.1%)	7107 (71.1%)	7184 (71.8%)
Hypertension	114,058 (83.1%)	8233 (82.3%)	8301 (83.0%)
Diuretics	45,756 (33.4%)	3337 (33.4%)	3299 (33.0%)
Chronic pulmonary disease	45,942 (33.5%)	3357 (33.6%)	3307 (33.1%)
Fibrates or omega-3	14,049 (10.2%)	991 (9.9%)	1041 (10.4%)
Ezetimibe	3575 (2.6%)	292 (2.9%)	237 (2.4%)
Severe hypoglycaemia	1947 (1.4%)	140 (1.4%)	151 (1.5%)
Systemic inflammatory disease	2768 (2.0%)	193 (1.9%)	207 (2.1%)
Renal complications	851 (0.6%)	67 (0.7%)	62 (0.6%)
Neurological complications	707 (0.5%)	59 (0.6%)	41 (0.4%)
4P-MACE	28,880 (21.1%)	2105 (21.1%)	2106 (21.1%)
Death (all causes)	9258 (6.7%)	680 (6.8%)	660 (6.6%)
Heart failure	7,374 (5.4%)	513 (5.1%)	569 (5.7%)
Infarction	8,746 (6.4%)	667 (6.7%)	661 (6.6%)
Stroke	5511 (4.0%)	392 (3.9%)	378 (3.8%)

Patient characteristics in the training, validation, and test sets are shown as count (percentage) for dichotomous variables, and as mean ± standard deviation for all others. Outcome prevalence is reported in the last five rows

### Model characteristics

The final deep learning model was based on 128 GRU units with ReLu activation, had no dropout at the recurrent layer level, but a recurrent dropout of 10%, and did not make use of a bias term in the attention mechanism.

### Discrimination capacity

Table 2 summarises the results of the primary and first secondary analyses. The proposed models yielded an excellent test set AUROC of 0.911 (95% CI 0.904–0.919) for 4P-MACE. The AUROCs for heart failure (0.807, 95% CI 0.790–0.824), myocardial infarction (0.811, 95% CI 0.795–0.826), and stroke (0.835, 95% CI 0.814–0.855) were also satisfactory. The AUROC for death (0.752, 95% CI 0.734–0.770), while lower, was also acceptable, and significantly better than random (0.5).

**Table 2:** Model discrimination performance

	RNN model (2D input: GLMs and time)		
Outcome	True sequence	Inverted sequence	Random sequence
4P-MACE	0.911 (0.904–0.919)	0.892 (0.883–0.900)*	0.905 (0.897–0.912)*
Heart failure	0.807 (0.790–0.824)	0.808 (0.790–0.826)	0.807 (0.789–0.824)
Myocardial infarction	0.811 (0.795–0.826)	0.799 (0.783–0.815)*	0.804 (0.789–0.819)*
Stroke	0.835 (0.814–0.855)	0.828 (0.808–0.848)	0.831 (0.810–0.852)
All-cause mortality	0.752 (0.734–0.770)	0.794 (0.777–0.811)*	0.777 (0.760–0.795)*

The table shows the AUROC of the proposed model on 4P-MACE and its four components on the test set (N = 10,000) when fed by the actual sequence of GLMs (second column), and an inverted and a randomised versions thereof (third and fourth columns). *p < 0.05 versus the true sequence

### Efficiency of sequence learning

The first secondary analysis highlighted that the overall 4P-MACE performance was very sensitive to any artificial alteration of the true order of GLMs: a random shuffle of trimester caused a statistically significant drop 0.006 points of AUROC, while a completely inverted ordering one of 0.019. We observed another distinct pattern for myocardial infarction, with drops of, respectively 0.007 and 0.012, and a similar but non-significant one for stroke (0.004 and 0.007). Heart failure classification performance appeared unaltered, while death exhibited the opposite phenomenon, with sequence alteration yielding counterintuitive, but highly unstable improvements.

### Performance over standard models

**Fig. 2.**
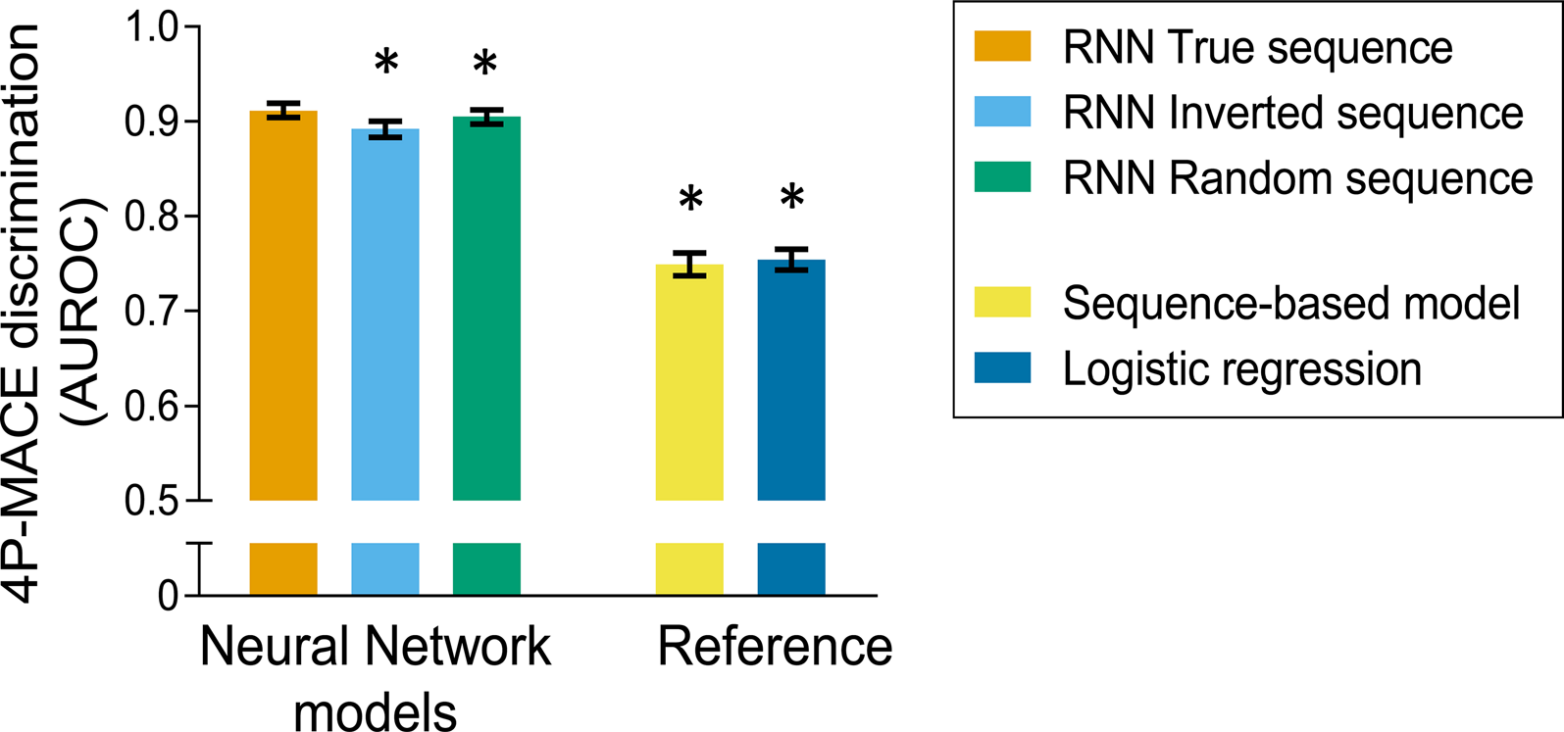
4P-MACE discrimination performance. The figure summarizes the area under ROC curves (AUROC) for the discrimination of 4P-MACE by the models shown in Tables 2 and 3. *p < 0.05 versus the RNN model with true GLM sequence

The second and third secondary analyses, focused on challenging the assumption that all three dimensions (timing, sequence, and GLM type) were useful for prediction, showed that neither the sequence-based model (AUROC 0.749, 95% CI 0.737–0.761), nor the bag-of-words logistic regression (0.754, 95% CI 0.743–0.765) could approach the 4P-MACE classification ability of the proposed RNN model (Table 3). In fact, the comparator models’ performance was almost superimposable and approximately 16% worse. The sequence-based model’s hyperparameters were: embedding size of 64, 64 GRU units, ReLu activation, 10% dropout and recurrent dropout. Figure 2 summarizes the discrimination performance in terms of AUROC of RNN models versus standard models.

**Table 3:** Comparison with standard models

Model	AUROC (4P-MACE)
RNN model (2D input: GLMs and time)	0.911 (0.904–0.919)
Sequence-based model (1D input: GLMs)	0.749 (0.737–0.761)*
Logistic regression (static input: GLM types)	0.754 (0.743–0.765)*

The table shows the AUROC of the proposed model on 4P-MACE on the test set (N = 10,000) compared to that of a sequence-based model and of a logistic regression on GLM types. *p < 0.05 versus RNN model

### Attention landscapes

**Fig. 3:**
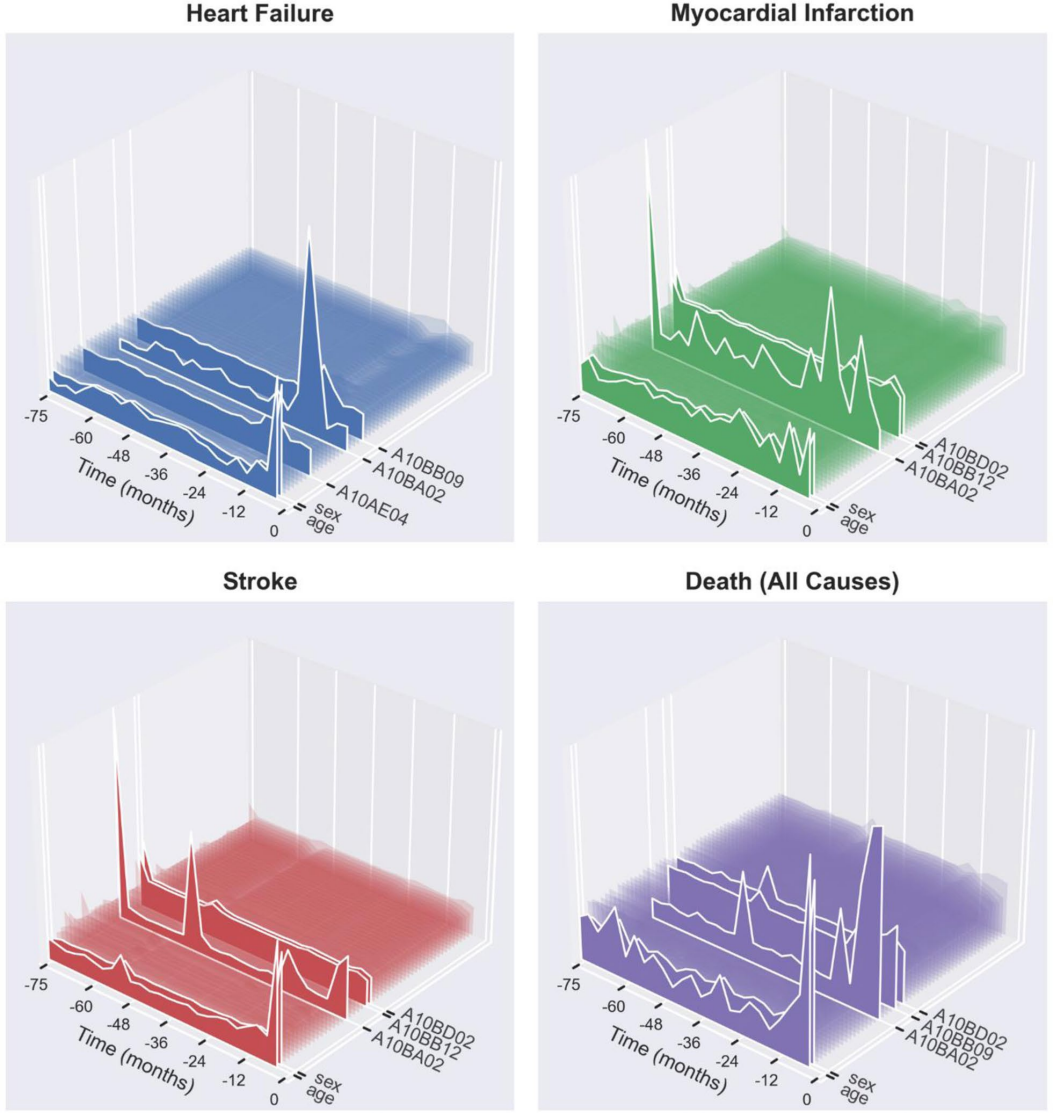
Attention landscapes associated with 4P-MACE components. Each panel shows the average attention profile associated with the respective outcome, normalised trimester by trimester. The X axis represents time in months as a negative offset to event or exit time; the Y axis represents the input variable (age, sex, diabetes duration, or GLM ATCs); the Z axis is the normalised average attention matrix across all training subjects. The variables with the most varied attention landscapes for each outcome are highlighted via solid polygons. A10BB09, gliclazide. A10BB12, glimepiride. A10BA02, metformin. A10BD02, metformin and sulfonylureas. A10AE04, insulin glargine

The average, renormalized attention landscapes for each of the four 4P-MACE components were qualitatively similar: they highlighted age and sex, and a small minority of GLMs as those presenting a consistent temporal pattern associated with 4P-MACE. Specifically, these were metformin (for all outcomes), combination of metformin and sulphonylureas (for myocardial infarction, stroke, and death), gliclazide (for heart failure and death), glimepiride (for myocardial infarction and stroke), and insulin glargine (for heart failure). With regards to the relevance of time, the attention landscape for heart failure highlighted the latest period of the observation interval as the most important for prediction. Myocardial infarction and stroke showed attention landscapes divided between early and late trimesters. The attention landscape for mortality was divided between the near past and the latest observations (Fig. 3).

## Discussion

In this study, we addressed the question of whether the detailed temporal trajectory of GLM in the patient’s history is associated with subsequent cardiovascular events, beyond the use of specific classes of drugs. To this end, we developed a new RNN model incorporating GLM sequence and temporal information, such as order, duration, and contemporaneity of treatments. It yielded an excellent capacity for discriminating MACE with > 91% AUROC in a test-set of 10,000 patients, i.e. after being developed and validated in completely separated cohorts of patients. For individual 4P-MACE components, discrimination was greater for heart failure and athero-thrombotic events than for all-cause mortality. This may be due to the fact that all-cause mortality can have several causes not reflected by GLMs and their time-resolved trajectory. We underline that, in this study, we used all-cause mortality in place of cardiovascular mortality as a MACE component because causes of death were not available in the database. Of note, in the last decade, cancer is taking over cardiovascular diseases as a cause of death among people with diabetes [20]. Therefore, all-cause mortality is likely less associated to the history of GLM among patients with diabetes.

We then evaluated whether the RNN model outperformed other models and which was the most important dimension driving improved discrimination capacity. First, we found that altering the GLM sequence led to a significantly worse 4P-MACE discrimination. This means that the true sequence of GLMs in the patient’s history has a substantial impact on the ability to identify patients with subsequent MACE, independently of other temporal features, such as duration and contemporaneity of treatments. Of note, exploding 4P-MACE components, it appears that the RNN model with the true GLM sequence outperformed RNN models with the inverted or random sequence for discriminating occurrence of myocardial infarction, while a paradoxical worse performance was observed for all-cause death. Reasons for this latter unexpected finding may be found in the competing risk issue or in the de-prescription of GLMs that occurs in some patients with very short life expectancy, for whom diabetes management is no longer a priority [21, 22].

Second, we tested to what extent other temporal dimensions contributed to the excellent discrimination capacity of the time-resolved RNN model. RNN models considering only the sequence of GLM without other temporal dimensions had dramatically worse 4P-MACE performance as did a logistic (non-RNN) model devoid of all temporal information, with an absolute ~ 16% lower AUROC (0.75 vs. 0.91). The difference was similar for individual components of the composite outcome, except for discrimination of all-cause mortality, which displayed no significant difference, likely for the reasons explained above.

The drop in performance resulting from discarding all the temporal information was substantially greater than that observed after only altering the GLM sequence. This leads to the speculation that features of the GLM trajectory unrelated to their order are more important in determining the outcome than the sequence itself. Therefore, it emerges that the GLM combination pattern and the duration of treatment are strongly associated with subsequent cardiovascular events. These features have important clinical implications. First, attention should be paid to combination therapies, as not all possible GLM combinations are rational and validated by dedicated trials. Second, choosing GLM regimens provided with greater durability could result in better outcomes, as this would imply a longer duration of treatment with the same regimen.

One typical issue when dealing with the outputs of machine learning approaches refers to the logical interpretation framework, i.e., the extent of extrapolation needed to derive clinical salience from the findings. Our analysis clearly shows that learning with time-resolved GLM data allows better discrimination of patients who experienced a subsequent MACE, but this approach is not suitable to dissect which are the GLM regimens or trajectories associated with lower or higher MACE rates. To gather further insight on this point, we incorporated attention maps into the RNN model. In image classification by artificial intelligence (a common example is Google lens), attention maps allow identifying elements of the image that are highlighted as helpful as compared to the background. In our model, the landscapes derived from averaged attention maps highlight therapies whose time-resolved trends are particularly linked to the outcomes. This is, to date, the best we can do to dissect components of the GLM trajectory that most contribute to discrimination. Interestingly, these therapies were metformin, sulphonylureas, and insulin glargine. Besides being the most common therapies for the management of T2D during the period of observation, they appear to be the major determinants of the RNN model’s ability to discriminate patients with subsequent MACE. Sulphonylureas and insulin have been repeatedly shown to be associated with adverse cardiovascular outcomes in several observational studies [23], though RCTs show these drugs may be considered safe from a cardiovascular standpoint when compared to placebo or to cardiovascular-neutral comparator [24–26]. However, none of prior observational studies explored the impact of the order, combination, and duration of treatment. We speculate that early initiation of sulphonylureas or insulin, or long treatment with the metformin/sulphonylurea fixed-ratio combination in the patient’s history is a major driver of the RNN model’s discrimination capacity toward MACE. Further studies will be needed to verify this point. On the other side, no attention was drawn to GLM known to be provided with cardiovascular protective effects, namely SGLT-2 inhibitors and GLP-1 receptor agonists. Although we have already shown the protective effects of such drugs in the same database [27–30], it is possible that a reverse causality association with 4P-MACE and the lack of patient matching for covariates diluted or nullified the evidence for lower MACE rates among users of these two drug classes. We herein do not want to challenge data on cardioprotective drugs, which were used by a small minority of patients as compared to metformin, sulphonylureas and basal insulin, limiting their contribution to the average attention landscape toward MACE. Repeating the same analysis with data updated to most recent prescription patterns might identify trajectories of newer drugs as relevant for outcome discrimination.

Another interesting observation from attention landscapes is related to the timing of attention, which differs for the type of 4P-MACE component. A difference was noted between discrimination of athero-thrombotic events and heart failure, with the latter being more influenced by the latest GLM pattern before the event. This may reflect the different pathophysiology of heart failure and the timing of its occurrence, as opposed to the slow progression of atherosclerosis.

Difficulty in determining the best GLM trajectories is a major limitation of this approach, along with its classification intent, which was not to predict future events as it could be done with other methods like Dynamic-DeepHit [31]. Further limitations of this study are intrinsic to the nature of the source data. In fact, the administrative database typically does not contain several relevant clinical-level information, such as body mass index, blood pressure, smoking status, glycaemic control, and lipid profile. Incorporation of all these time-varying factors, together with the availability of wider observation intervals (the current version of our model is limited to approximately 6 years of input data) may modify the relative importance of the GLM patterns. Future work in this direction may include extending the same experimental framework to a wider array of drugs, such as lipid-lowering, anti-platelet, and anti-hypertensive agents; and identifying a suitable modelling technique to highlight specific patterns of usage and their positive or negative correlation with 4P-MACE. This would allow evaluating the interaction between GLM trajectories and other medications typically used by people with T2D.

## Conclusion

In summary, this novel approach of classification by a deep RNN model with attention landscapes reveals the importance of the detailed patient’s trajectory of GLM use over time in discriminating subsequent occurrence of a 4P-MACE and highlights some drugs driving the discrimination. While further data analytics will be needed to better calculate the best treatment trajectories, from a clinical perspective, these findings reinforce the concept that the patient’s past GLM history can impact future cardiovascular outcomes.

## Data Availability

Restrictions apply to data analysed in this study. Aggregate information and the source code to the models are available from the corresponding author at a reasonable question.

## Abbreviations

ADAM: A method for stochastic optimization
ATC: Anatomic therapeutic classification
AUROC: Area under receiver characteristic curve
GLM: Glucose lowering medications
GRU: Gated recurrent unit
ICD: International classification of diseases
LSTM: Long short-term memory
MACE: Major adverse cardiovascular events
RNN: Recurrent neural network
T2D: Type 2 diabetes
